# Ulcerative Diseases of the Mouth

**Published:** 1888-09

**Authors:** George J. Dennis

**Affiliations:** St. Louis, Mo.


ARTICLE III.

### ULCERATIVE DISEASES OF THE MOUTH.

BY GEORGE J. DENNIS, M. D., D. D. S., ST. LOUIS, MO.

[Read before the Missouri State Dental Society, July 10, 1888.]

Superficial solution of the continuity of the external tissues of the body with the exudation of pus, lymph, and degenerated cells, are among the frequent diseases of the body. Mucous membranes are especially liable to degeneration and ulceration, owing to their delicate organization and the fact that they are more abundantly supplied with follicles and glands of greater or less secreting power, which easily become occluded or become inflamed and result in ulcerations of various forms. That lining of the mouth is very liable to such inflammations and degenerations, exposed as it is to irritations external as well as to those sympathetic irritations, dependent upon irritation in other parts of the alimentary tract. Hence, we find in the various complications arising from gastritis, enteritis, and other inflammations of the nutrient canal, the mouth also becomes an important factor. Constitutional diseases frequently have their local manifestations in the mouth which often end in extensive ulcerative processes.

As in other parts of the body, ulcerations take on a variety of appearances of greater or less diagnostic value. They may be round, oval, or irregular; their edges smooth, or ragged; their sides sloping, perpendicular, or undermined; their bases soft, and extensively infiltrated, or the infiltration may be hard and circumscribed, or very rarely, there may be no infiltrations whatever.

The floors of these ulcers may be sensitive and extremely painful, dependent upon the irritation, seat of the disease, and the acuteness of the attack.

The simplest ulcers found in the mouth, and those most frequently seen, are aphthæ. They appear in small round, or oval patches, in adults generally on the lips, at the reflection of the mucous membrane or to the gums. In children they are more apt to be found on the palate. Their history is interesting. From irritations, mechanical or chemical, from acid secretions of the mouth, from perversion of nutrition, or of the gastro-intestinal secretions, or from debilitating surroundings rendering the patient susceptible to disease, inflammation is set up, catarrhal in form at first, with heat, redness, and slight swelling of the part. Later, the follicles of the mucous membrane become inflamed, little elevations containing pits appear on the surface. The mucous membrane becomes thickened by infiltration and in a few hours a whitish exudate is poured out in round, or oval spots, which vary in size, from that of a mustard seed to that of a bean. They are more often isolated, but may run into each other giving an irregular appearance.

The inflammation continuing there is quickly an increased secretion from the mucous membrane with cellular infiltration of the underlying tissue. The whitish exudate breaks down, degenerates, and the epithelial covering is thrown off leaving little whitish, yellow looking ulcers. Extreme sensitiveness of these spots on movement of the parts, accompanied by an avoidance of solid food, usually a lack of appetite and a feverish state of the system are characteristic of aphthæ. A slight odor may accompany these changes, but it is never so intense as in the severer ulcerative diseases. They remain, if not treated, two or three weeks, and again may heal in a few days on removal of the irritant.

The process of healing begins in a reddening of the floor of the ulcer, granulations covered with epithelium are thrown up from the bottom, and in a few days the entire ulcer has healed leaving no cicatrix.

When diphtheria is prevalent aphthæ may at first simulate it. However, in diphtheria the exudate begins on the tonsils and pharynx, in a thin translucent film looking like the white of an egg, works forward, growing thicker and more extensive, and springing from little centers as it spreads. The constitutional symptoms in diphtheria are early very marked and preclude any error in diagnosis.

Another ulcerative process is stomacace. It begins as an inflammation of the margins of the gums which quickly passes into destructive ulceration. The margins of the gums about the incisor teeth are its favorite point of commencement, then extending posteriorly to the molars. It is peculiar in that only one side is affected at a time. Later in its course it attacks the membrane lining the cheeks and tongue, but leaves the hard and soft palates entirely exempt from its destructive ravages. Accompanying these changes is an extensive redness and swelling and most offensive odor. Hæmorrhages occur spontaneously. The secretions of the salivary and mucous glands are greatly increased and the saliva is changed in color to a brownish red. When this train of symptoms is completed in the adult the marginal ulcers penetrate deeper and deeper; the roots of the teeth are soon denuded and by the extensive destruction of tissue drop out; the pain at the same time being so great that life is a miserable existence. This disease seems by preference to select children who have just completed their first dentition, while it often attacks soldiers in garrison. The causes ascribed are contagion and bad hygienic conditions. According to a noted writer these same symptoms may be those of ptyalism, and cites mercury as one of the principal causes in the adult.

Scorbutus is a disease whose ulcerative processes in the mouth are most extensive. It presents many of the symptoms of the former disease; the process ending in both with loosening and in the majority of the cases with the loss of the teeth. This difference is noted. In scorbutus the swelling of the gums is much greater, rising sometimes above the crowns of the teeth. Scorbutus is a constitutional disease, the ulceration of the mouth being simply symptomatic while stomacace is idiopathic in nature.

The syphilitic ulcers appearing in the mouth are chiefly secondary. Very rarely are they found primarily as the initial lesion. Their seat is on the pharynx, soft palate and tonsils. As the disease increases in severity they extend to the sides of the tongue, but never to the buccal surfaces. They grow slowly, are very permanent and stable in their location. They have also a great tendency to perforation, leaving behind great ulcers with ragged edges, and hard, callous bases. Only slight hæmorrhages occur, and the pain is so slight that the patient experiences comparatively little annoyance. Constitutional treatment quickly confirms the diagnosis as they yield in a short time to specifics.

Thrush is a disease of childhood belonging properly to the fungoid affections, but owing to the tendency of the mucous membrane to ulceration may be considered here.

The fungi present in thrush are not the cause of the disease, but simply one of the effects, and are found only in unhealthy mouths where the secretions are vitiated, acid, and the mouth already subject to inflammatory action. As in the other ulcerative diseases the health of the patient is usually poor and the hygienic surroundings very bad.

The fungi once finding a ready soil in which to grow, set up a higher grade of inflammation, leading to destruction of the epithelial tissue. As the disease progresses the sub-epithelial tissue may become involved and the epithelial patches covered with the fungi fall off.

On examination the loosened masses are found to consist of mucus, epithelial cells, pus, and spores, held together by the long slender filaments of the oidium albicans.

This disease may extend to the nasal passages and pharynx, and has even been found throughout the full extent of the alimentary canal. It is usually found in emaciated children; very rarely is it found in healthy persons. The symptoms are at first those of ordinary stomatitis, increased by heat, redness and swelling, pain on movement of the parts, a longing for cold drinks, a greatly increased amount of saliva and marked gastro-intestinal disturbance. A distinctly acid reaction of the mouth, together with the white patches, make the diagnosis easy.

So rarely is the oidium albicans found in the mouth of a healthy adult person, the following case treated by Dr. B. Merrill Ricketts, of Cincinnati, Ohio, with whom I had the pleasure of seeing the patient, may prove interesting. I give his paper as read before the Cincinnati Medical Society, March 1, 1887.

"H. T. L., physician, æt. 30, fairly nourished, habits good, bowels regular, subject to sick headache every three or four weeks, inveterate smoker (two cigars daily.)

About August 1st, 1886, noticed that tongue and roof of mouth was sore. Supposed it was due to smoking a pipe; examination showed several white patches on mucous membrane of hard palate, one on the apex, and one on the base of the tongue.

They were irregular in outline and but slightly elevated. They were also in close apposition with the epithelium, the papillæ being exposed where they were torn loose.

The slight congestion accompanying these patches caused a burning sensation.

Stopped smoking and applied solution of boric acid for one week, but no improvement; changed treatment to application of tannic glycerate for one week. Condition continued to grow worse. Solution (1.500) of bichloride of mercury was then applied for a few days without any change. A five per cent. solution of argenti nitras was then applied for three weeks. Condition at the end of this time very much worse.

There was now inability to masticate solid food, owing to the excessive soreness of the gum and palate. Careful examination revealed the presence of another aphthous patch upon the tongue—making in all three—each as large as a nickel, and a large patch on the gums at either angle of the lower jaw, two small patches on lower lip and several nodular patches on each cheek.

The secretions of the mouth gave a neutral reaction.

Whenever the mucous membrane became abraided, the fungi lost no time in attacking and developing upon it, they having the faculty of reproducing themselves upon being detached.

It is no wonder that the mouth should be a favorable location for parasites when we consider that it is the principal channel through which food, air and water enter the body, especially when the secretion are abnormal or there is some gastric disturbance. The normal saliva being virtually a parasiticide does not allow them to enter unmolested.

Mr. Gruby, in 1852, was the first to show the true nature of this fungus. It being named by him *Aphthaphyte or Cryptogame du muguet.* It was afterwards called by Robin *Oidium Albacans* and referred to the genus Oidium. ("Hist. Nat. des Vig. Parasit," 1853.)

Mr. Clark says, (Disease of Tongue, page 85, London, 1873,) 'that it forms delicate horizontal filaments which are apparently homogeneous in structure and from which short articulated pedicles take their rise. The uppermost cells of these pedicles become expanded into oval bodies which fall off, germinate and become new filaments. It is generally found growing in tangled masses like minute bunches of mistletoe, mixed with the debris of scattered spores, cells of leptothrix and epithelial scales.'

About September 1st (or five weeks from the time it was first noticed) the treatment having failed to give the least benefit whatever, I decided to resort to some form of treatment that would be more heroic. This was done by applying the solid stick of nitrate of silver every other day, or as often as was necessary, to keep up a continuous sloughing of the mucous membrane and all parasites that it might reach.

Within a week there was a marked change for the better in all patches, except those which were nodular and somewhat elevated upon the cheeks.

These were removed with the scissors and their remaining cavities thoroughly cauterized.

So far there has been no return of those treated in this way.

There have been times when there was not existing any slough, but the surface treated with the caustic was not allowed to become entirely healed.

Improvements has continued until there is now to be seen but two very small patches upon the palate, each near the posterior molar and one at either angle of the jaw.

The one upon the apex of the tongue is almost indistinct, not (as might be supposed) due to any application of the stick caustic, but to coming in contact with the palate after it had been treated with the caustic.

During all this time the patient has paid strict attention to his diet. He being a physician could more fully appreciate the importance of what might otherwise be considered valueless in the treatment of such an obstinate disease."—*Ohio Dental Journal.*

